# Metabolism, toxicity and anticancer activities of arsenic compounds

**DOI:** 10.18632/oncotarget.14733

**Published:** 2017-01-18

**Authors:** Islam Khairul, Qian Qian Wang, Yu Han Jiang, Chao Wang, Hua Naranmandura

**Affiliations:** ^1^ Department of Toxicology, School of Medicine and Public Health, Zhejiang University, Hangzhou, China; ^2^ College of Pharmaceutical Sciences, Zhejiang University, Hangzhou, China; ^3^ Ocean College, Zhejiang University, Hangzhou, China

**Keywords:** arsenite, acute promyelocytic leukemia

## Abstract

A variety of studies indicated that inorganic arsenic and its methylated metabolites have paradoxical effects, namely, carcinogenic and anticancer effects. Epidemiological studies have shown that long term exposure to arsenic can increase the risk of cancers of lung, skin or bladder in man, which is probably associated with the arsenic metabolism. In fact, the enzymatic conversion of inorganic arsenic by Arsenic (+3 oxidation state) methyltransferase (AS3MT) to mono- and dimethylated arsenic species has long been considered as a major route for detoxification. However, several studies have also indicated that biomethylation of inorganic arsenic, particularly the production of trivalent methylated metabolites, is a process that activates arsenic as a toxin and a carcinogen. On the other hand, arsenic trioxide (As_2_O_3_) has recently been recognized as one of the most effective drugs for the treatment of APL. However, elaboration of the cytotoxic mechanisms of arsenic and its methylated metabolites in eradicating cancer is sorely lacking. To provide a deeper understanding of the toxicity and carcinogenicity along with them use of arsenic in chemotherapy, caution is required considering the poor understanding of its various mechanisms of exerting toxicity. Thereby, in this review, we have focused on arsenic metabolic pathway, the roles of the methylated arsenic metabolites in toxicity and in the therapeutic efficacy for the treatments of solid tumors, APL and/or non-APL malignancies.

## INTRODUCTION

Arsenic is one of the most notorious poisons since the ancient times. Humans are exposed to arsenic predominantly through drinking water, air, food, occupation and other environmental sources [1–3]. Especially, arsenic present in seafood is frequently as organic forms (i.e., arsenobetaine, arsenosugars, etc.), which is considered to be nontoxic or much less toxic than inorganic arsenic. [4–6] In fact, inorganic arsenic (e.g., arsenate and arsenite) is the most poisonous form which is found in the contaminated drinking water in many parts of the world [7, 8], and there are at least 40 countries in the world with arsenic concentrations in ground water are higher than 10μg/L. Importantly, it has been estimated that 200 million people in the world are at risk from health effects associated with high concentrations of arsenic in their drinking water [9–10].

As a consequence of the biggest arsenic calamity has emerged in several parts of the world, the situation of arsenic toxicity is alarming and severe health problems are reported amongst the inhabitants relying on ground water as sources of drinking water [9]. Every year, new areas are being contaminated and identified. The use of ground water for irrigation and the bioavailability of arsenic to food crops and the uptake by humans and livestock through the food chain have opened additional pathways for arsenic exposure all over the world. The magnitude of the arsenic catastrophe has projected to be the largest in history of environmental disaster that will be more serious than those at Chernobyl, Ukraine in 1986 and Bhopal, India in 1984 [11–13]. Long-term or chronic exposure to arsenic is related to severe adverse health effects including dermatitis, cardiovascular diseases, diabetes mellitus, chronic bronchitis, immune disorders, peripheral neuropathy, liver damage, renal failure, adverse reproductive outcomes, hematological effects, and other ailments [14–21]. In fact, arsenic affects almost all vital organs of human body causing the damage or dysfunction.

**Figure 1:**
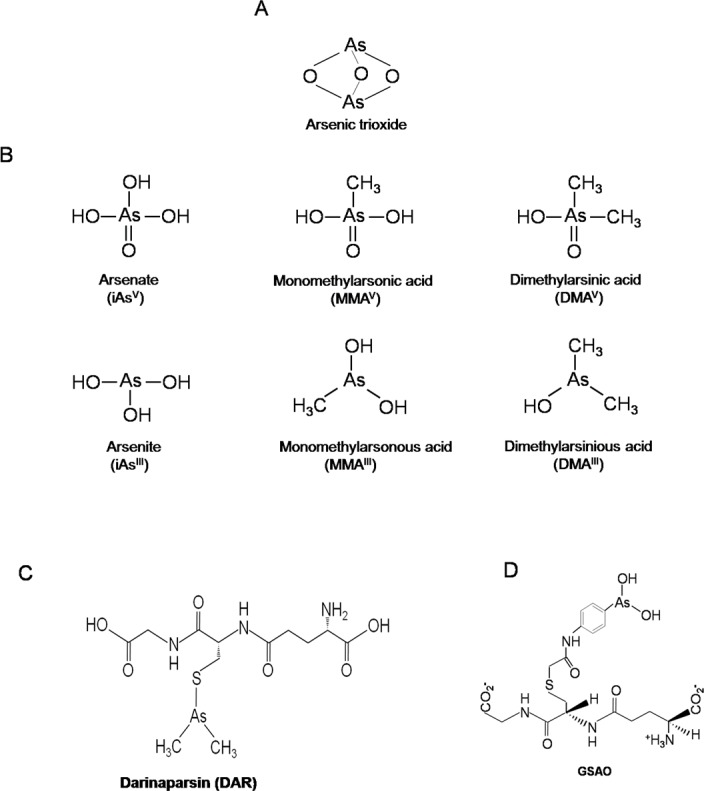
Structures of Different Arsenic Compounds **A**. Structures of As_2_O_3_, or **B**. two major forms of inorganic arsenics namely; iAs^V^, iAs^III^ and major methylated metabolites including MMA^III^, DMA^III^, MMA^V^ and DMA^V^. **C**. DAR and **D**. GSAO.

Along with the adverse health effects, social and economical problem also occurs for the population of the arsenic affected countries. On the other hand, arsenic trioxide (As_2_O_3_) has been used as a drug for the treatment of various diseases in ancient China and Greece for over 3000 years [22]. In addition, As_2_O_3_ (Figure 1A) has recently shown remarkable therapeutic efficacy in patients with acute promyelocytic leukemia (APL) [23]. Though As_2_O_3_ has shown excellent efficacy for the treatment of APL, however, as a single-agent, it could not provide satisfactory outcomes for the management of different solid tumors [24]. Recently, it has been reported that some organic arsenic species have strong anticancer effect against solid tumors. Thus, it is very important and need to be considered the arsenic metabolism pathway and its organic metabolites for the treatment of cancers. In addition, it would be helpful in elucidating the probable role of different arsenic species (including inorganic arsenic and its methylated metabolites) in relation to toxicities along with the uses of arsenic as a drug. Especially, the roles of the methylated metabolites in the therapeutic efficacy and toxicity are still indistinct and needs to be elucidated. Thereby, in this review, we have focused on arsenic metabolic pathways, the roles of the methylated arsenic metabolites in toxicity and in the therapeutic efficacy for the treatments of solid tumors, APL and/or non-APL malignancies. Our findings will provide a new and better understanding of arsenic toxicity and therapeutics, which will further accelerate basic understanding on the mechanism of arsenic-induced diseases and the probable role of its active trivalent intermediate metabolites in the treatment of numerous neoplastic diseases.

## INFLUENCES OF ARSENIC POTENTIAL FOR TOXICITY AND AS ANTICANCER AGENTS

### Metabolism of arsenic

Epidemiological studies indicate that iAs^III^ methylation is incomplete and the composition of arsenic metabolites in urine varies from person to person though they were exposed to the same level of arsenic in drinking water [25]. Interindividual differences in capacity to produce methylated arsenicals are linked to the differences in susceptibility to arsenic-induced diseases [26]. The distribution of the different arsenic species depends on many factors, including the species of arsenic administered, species of the plant or animal, different types of cells within the same organism, the availability of glutathione (GSH) and other biomolecules containing thiol (−SH) groups, the principle arsenic methylating enzymes, and on the pH of the matrix [27, 28]. Generally liver is the primary target organ for the metabolism of arsenic [29, 30]. It has been demonstrated that inorganic arsenic are commonly methylated in liver in the presence of a methyl donor S-adenosylmethionine (SAM) and a co-factor glutathione (GSH) with arsenicmethyltransferase (AS3MT) to relevant monomethylated [e.g., monomethylarsonous acid (MMA^III^) monomethylarsonic acid (MMA^V^)] and dimethylated arsenic metabolites [e.g., dimethylarsinous acid (DMA^III^), dimethylarsinic acid (DMA^V^)], and finally excreted into urine (Figure 1B). However, *in vitro* study has also indicated that other tissues seem to have arsenic methylating capacity and the highest amount of arsenic methylating activity is in the cytosol of testes, followed by kidney, liver, and lungs [31, 32]. The tissue distribution and retention of arsenic depends on the bioconversion of methylated metabolites and determines its actions as a toxicant or carcinogen [33].

The chemical plausibility of different methylation schemes has been recently evaluated and questioned [33]. The pioneer work of Frederick Challenger and his colleagues first described a sequence of alternating reactions in microorganisms known as the reductive/oxidative methylation pathway, during this process, iAs^V^ is first reduced to the more toxic iAs^III^, and then methylated and oxidized to MMA^V^ simultaneously [34, 35]. Then MMA^V^ get reduced to MMA^III^ and during oxidative methylation it is transformed into DMA^V^, which is then reduced to DMA^III^. In certain species, but not in humans, during oxidative methylation DMA^V^ then can be converted into trimethylarsine oxide (TMAO^V^) [36]. Due to the lack of sufficient analytical tools, Challenger was not able to identify any of the postulated intermediate species of the pathway, later Cullen and co-workers identified methylated intermediates to provide further evidence supporting the Challenger pathway in fungal and algal cultures [33].

Obviously, Challenger and his colleagues open up and made the basement of arsenic methylation pathway, however, alternative schemes for arsenic methylation suggesting glutathione- or protein-conjugated intermediates have also been proposed. Hayakawa et. al., have postulated that iAs^III^ species persist during methylation reactions and that oxidation to pentavalent arsenic species occurs (somehow) after methylation. In this pathway, they proposed that the arsenic-glutathione complexes are the major substrates for AS3MT, and that the yielding of inorganic arsenic triglutathione iAs^III^(GS)_3_ can be further methylated to form the monomethylarsonic diglutathione MMA^III^(GS)_2_ and dimethylarsinic glutathione DMA^III^(GS) in the trivalent states in the presence of methyl-group donor SAM and GSH [37]. The main limitation of this method is that, how does CH_3_^−^ group leave a positive center?

William R. Cullen [33] interpreted enzymatically catalyzed methylation according to the Challenger pathway and successfully solve the problem related with Hayakawa et. al. postulated pathway. In this pathway, protein or exogenous thiol reactants would neutralize the charge on arsenic by providing one more thiolate to the arsonium center and neutralize the charge on arsenic. Resulting an opportunity for reductive removal of a disulfide and leave the protein bound trivalent methylarsenic species.

**Figure 2:**
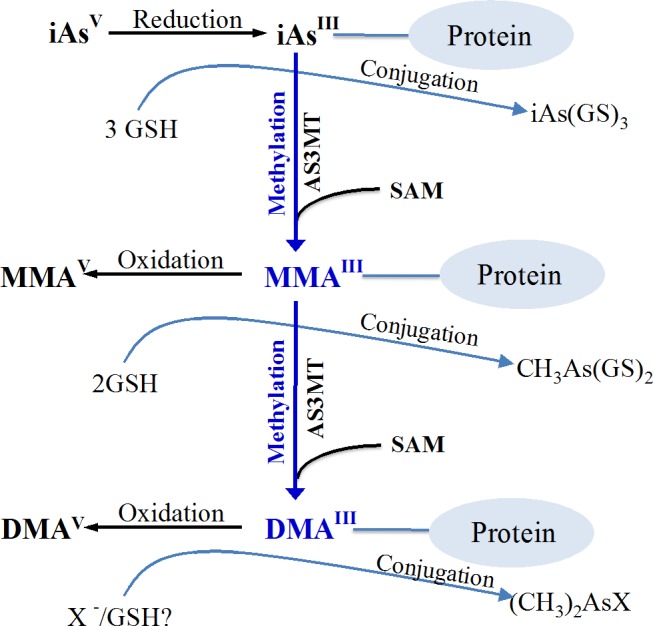
Proposed Arsenic Metabolism Pathway in Human In the body, the inorganic trivalent arsenic is metabolized in a protein-bound form having successive reductive methylation by arsenicmethylatransferase (AS3MT) in the presence of glutathione and S-adenosylmethionine (SAM). In particular, monomethylarsonous acid (MMA^III^), dimethylarsinous acid (DMA^III^) are the trivalent intermediate metabolites which in protein-bound form are further oxidized to corresponding pentavalent monomethylarsonic acid (MMA^V^) and dimethylarsinic acid (DMA^V^), which are considered to be less toxic end products of arsenic metabolism.

Kala and coworkers have reported that arsenic-GSH complexes [e.g., iAs^III^(GS)_3_, MMA^III^(GS)_2_ and DMA^III^(GS)] were predominantly found in bile and excreted into the bile through the multidrug resistance protein 2/canalicular multispecific organic anion transporter (MRP2/cMOAT) [38]. Moreover, we proposed that reductive methylation is the heart of an alternative pathway (Figure 2). In this pathway, iAs^III^ is metabolized in the body bound to proteins in a trivalent form during the successive reductive methylation by AS3MT in the presence of GSH and SAM, then oxidized to corresponding pentavalent arsenic species [39].

**Figure 3:**
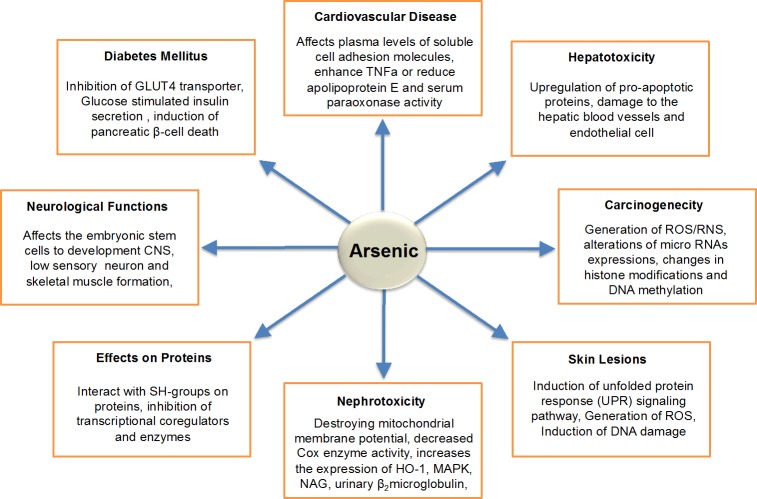
Schematic Diagram Represents the Toxicities of Arsenic

Wang et al. [40] showed the effects of different reductants such as GSH, cysteine, and tris(2-carboxyethyl)phosphine (TCEP) on the kinetics of arsenic methylation catalyzed by hAS3MT. They suggested that the valence state of arsenic is unchanged during the methylation reaction occurs on hAS3MT. Reductant helps to exposing the active sites of hAS3MT for binding to iAs^III^ by reducing the disulfide bond between the cysteine residues of enzyme and enzyme-AdoMet-reductant. In addition, our group along with other groups found that the trivalent arsenic species are mostly in the protein-binding forms, indicating arsenic possess higher affinities to bind with cysteine rich proteins than to glutathione [39, 41, 42].

Arsenic cytotoxicity is completely dependent on its oxidation state and chemical structure. During the detoxification of iAs^III^, the compound is biomethylated to mono- and dimethylated (in some cases trimethylated) arsenicals of varying toxicity. Some of these metabolites will be less while others will be more toxic than iAs^III^. For instance, pentavalent methylated arsenicals are less toxic, while trivalent methylated species are highly cytotoxic and genotoxic than its precursor iAs^III^ [43]. Notably, methylation of arsenic is recently considered to be a bioactivation and detoxification pathways, but it seems to depend on the condition of arsenic exposure and its levels. For example, in case of chronic exposure of arsenic, it may be bioactivation process, but in others (including a single low dose and acute exposure), it may be detoxification pathway. The overall toxicity of arsenic is dependent on the rate of methylation of the iAs^III^ formed. Many *in vitro* studies have shown that the trivalent forms of MMA^III^ and DMA^III^ are much more toxic than the pentavalent forms, and trivalent methylated arsenicals are in particular more toxic than trivalent inorganic arsenic [44–46]. Moreover, it has been found that MMA^III^ and DMA^III^ can inhibit the activities of many enzymes and are more genotoxic than iAs^III^ [47–50]. Styblo et al. [43] reported that MMA^III^ was highly toxic to human cells (e.g., hepatocytes, epidermal keratocytes, and bronchial epithelial cells) compared to inorganic species.

Recent study has indicated that higher proportions of methylated arseniclas in urine of arsenic exposed population are the evidence of increased risks of skin and bladder cancer [51–53]. Depending on the improvement of analytical techniques, toxicity studies of arsenic gradually changes from oxygenated metabolites of inorganic arsenic to thiolated arsenic metabolites, and many studies found that thiolation can change arsenic toxicity. The thiolated arsenicals are readily taken up by bladder cells and quickly converted to the corresponding trivalent oxygenated form of arsenic and showed their toxicities [54, 55].

On the other hand, the anticancer effects of arsenic are not only from direct or indirect influences on the genetic levels, but also strongly related with the distinctive arsenic metabolism pathways. AS3MT catalyzes arsenic to methylated products, by utilizing S-adenosyl methionine (SAM) which is an important co-factor of DNMTs and producing SAH, resulting to bring about the DNA hypomethylation [56]. Therefore, arsenic may play a crucial role in the regulation of tumor suppressor genes by interfering with the DNA methylation patterns. More importantly, a few clinical studies have shown that different methylated arsenic species (e.g., MMA^V^, MMA^III^, DMA^V^ and DMA^III^) were found in blood, saliva or urine of APL patients after receiving arsenic treatment [57, 58]. Drobna et al., 2009 reported that As_2_O_3_ methylates to mono-methylarsenic (MMA) and dimethylarsenic (DMA) in the liver of APL patients [59]. Arsenic methylated metabolites are also found in hepatoma (HepG2) and glioblastoma cell lines (U87MG) [60, 61]. One group reported that As_2_O_3_ is metabolized into the methylated metabolites in the NB4 cell and argue that these intracellular methylated arsenic metabolites induce DNA hypomethylation and take part in the therapeutic effects of As_2_O_3_ in APL. Thereby, we should have to consider the importance of arsenic metabolism and its metabolites for elucidating the probable role of different arsenic species (i.e., including precursor and its metabolites) in relation to toxicities as well as in therapeutic efficacies [34, 40, 62].

### Arsenic uptake and transport

Arsenic as toxic agents or drugs, it needs to be accumulated in cells and exert the biological functions. Thus, arsenic toxicity is suggested to be due to its chemical species, uptake and efflux [63–65]. Among the different arsenic species, it has been identified that trivalent arsenic species are much more toxic than that of corresponding pentavalent arsenicals. Hence, to understand the role of arsenic, it seems to be essential to precisely comprehend the importance of influx and transporter pathways.

Aquaglyceroporins (AQPs) have been recognized as vital family of membrane proteins for the uptake of arsenic [66], and the most important members such as AQP3, 7 and 9 are found to be involved in the uptake of iAs^III^ into the cells [67]. It has been reported that AQPs mainly expressed in majority of the organs including liver, spleen, lung, adipose tissues and kidney [68]. Interestingly, AQP9 have also been identified to play an important role for uptake of organic methylated MMA^III^ [69]. Additionally, AQP9 was found to be highly expressed in the leukemic cell lines (e.g., NB4 cells), making the cell more sensitive towards arsenic treatment [69]. The down regulation of the AQPs in the cancer cells has shown to decrease the relative effectiveness of arsenic treatment [56]. For example, the chronic myeloid leukemia cell line (K562) is recognized less sensitive towards iAs^III^ treatment because of lacking adequate expression of AQP9 [69]. Moreover, when AQP3 gene was knocked down in human lung adenocarcinoma cell line (CL3), it was found that the CL3 cells became more resistant to iAs^III^ [70], indicating the AQPs are at least involved in cellular susceptibility towards iAs^III^.

On the other hand, for the efflux of trivalent arsenicals, human multidrug resistance proteins (e.g. MRP1, 2/ABCC1, 2) have been identified as essential transporters [71]. The MRP family contains nine ATP-binding cassette transporters (ABC) and is known for the efflux of drugs from the cells [72]. MRPs are capable of facilitating the transport of numerous conjugates of drugs or metals with sulfate, glucuronide and glutathione [73–75]. Regarding the arsenic efflux, it has been reported that both MRP1 and 2 along with the phase II conjugating enzymes like glutathione transferase (GST) are the major contributors for the resistance towards specific metals and toxicants [71, 76]. Few studies have shown that GSH is required for formation of arsenic-Glutathione conjugates [i.e., iAsIII(GS)_3_, MMAIII(GS)_2_] and then transport by MRP1/2 transporters into bile [77, 78]. More importantly, the expression of MRP1 protein has been found in various tissues, nevertheless, the expression of MRP1 in tumor and cancer cells is thought to render them less sensitive towards arsenic treatment [73]. Similar results were obtained from the AR2 cells (which are derived from NB4 cells with highly expressed MRP1) or HeLa-MRP1 transfected cells, these cells showed much resistance to As_2_O_3_ when compared with parental NB4 Cells or HeLa cells, respectively [79], suggesting that MRP1 may contribute to cellular resistance to arsenicals.

### Binding affinity of arsenicals to proteins

The properties of arsenic binding to proteins are one of the most important factors that have been found critical in influencing the sensitivity and resistance of a specie towards arsenic exposure. To date, it has been found that trivalent arsenic is able to inhibit more than two hundred enzymes. MMA^III^ has shown to display greater toxicity and/or carcinogenic potential than iAs^III^. Generally speaking, trivalent arsenicals have high binding affinity to sulfhydryl groups of proteins, while pentavalent arsenicals found to be not binding with sulfhydryl groups of proteins [80]. In addition, interaction with zinc finger proteins is considered to be an important mechanism of arsenic toxicity and carcinogenesis. Recent study has shown that MMA^III^ acts more effectively than iAs^III^ to destroy the normal integrity of zinc fingers.

Among the three arsenic trivalent species (i.e., iAs^III^, MMA^III^ and DMA^III^), our previous study have shown that MMA^III^ have much stronger binding affinity than other two species *in vivo* and *in vitro* [39, 81] because MMA^III^ binds with two closely spaced cysteine residues or dithiol cofactors. Contrast to MMA^III^, iAs^III^ needs at least three symmetrical cysteine residues in protein, indicating that MMA^III^ can easily bind with protein compared to iAs^III^ [82, 83]. On the other hand, DMA^III^ would have a weaker bond with cysteine residues compared to the cysteine complexes with MMA^III^ and iAs^III^ because the methyl groups change the electron density around the arsenic [84].

AS3MT, a cysteine rich enzyme that can catalyzes the biomethylation of arsenic *in vivo* and *in vitro*. Identifying the As-binding site in the AS3MT would facilitate understanding of the process by which the arsenic binds to AS3MT. In each arsenic species, for example iAs^III^ can bind to three, methylarsenite (MMA^III^) can bind to two, and dimethylarsenite (DMA^III^) can bind to one cysteine residues, respectively. It has been found that Cys156 and Cys206 in *h*AS3MT have been shown to be binding sites for As and recently Li et al. suggested Cys61 as the third binding site of iAs^III^ [85].

Interestingly, it has also been recognized that DMA^III^ forms a stable complex with hemoglobin of rats, rendering the animal tolerant to arsenic having minimal risk for induced toxicity with maximum arsenic accumulation [41, 86]. However, hamsters have been known to be very sensitive similar to human towards arsenicals, and found that arsenic is mostly distributed in organs of hamster rather being accumulated in red blood cells (RBCs) [86]. Due to the absence of cysteine (Cys) residue (Cys-13) in the alpha chain of hemoglobin in hamster or humans, dimethylated arsenic (DMA^III^) could not binds with hemoglobin, results in redistributed and accumulated in various organs [34].

## TOXICITIES OF ARSENIC

Different arsenic metabolites have different toxicities and the extent of toxicity is depends on the animal species, cell types and uptake rate of each arsenic compounds. For example, IC_50_ values of the same arsenic species are differ from cells to cells, in human hepatocytes is DMA^III^(GS) ≈ iAs^III^ << MMA^III^, while in human keratinocytes it is iAs^III^ << DMA^III^(GS) < MMA^III^, and in human bronchial cells it is DMA^III^(GS) < iAs^III^ ≈ MMA^III^ [43]. Recently a study carried out on wild type mice stated that the majority of iAs and methylated As species were found in liver as well as in pancreas indicating the adverse effects of iAs [87]. In generally, trivalent arsenicals are much more toxic than pentavalent counterparts and the reason behind is the lower rate of accumulation of pentavalent arsenic species [43, 88, 89].

On the other hand, arsenic toxicity *in vivo*, it has been reported that acute arsenic poisoning is associated with nausea, vomiting, abdominal pain, and severe diarrhea. Conversely, chronic ingestion of arsenite through contaminated drinking water results in the accumulation of arsenic in vital organs and act as the incidence for diabetes, atherosclerosis, hypertension, ischemic heart diseases, hepatotoxicity, nephrotoxicity, and cancer of the skin, bladder, and lungs. However, the actual mechanism by which arsenical induced diseases has not yet been fully elucidated. The present review importantly discussed the pathogenesis of arsenic-induced toxicity and end organ damage.

### Arsenic-induced diabetes mellitus

The trivalent arsenic species (i.e., iAs^III^, MMA^III^ and DMA^III^) have found to inhibit GLUT4 recruitment to the plasma membrane of insulin stimulated adipocytes by inhibiting the activity of 3-phosphoinositide-dependent kinase-I (PDK-1) and mammalian target of rapamycin (rictor-mTOR). Activity of PDK-1 and rictor-mTOR are necessary for the phosphorylation of Akt [90].

In fact, Akt is necessary for GLUT4 translocation and glucose transport. Therefore, loss of dual phosphorylation of Akt due to the effects of arsenic and its metabolites, causes significant inhibition of insulin-dependent glucose uptake and hence results in hyperglycemia [90]. Another group suggested that the insulin producing pancreatic β-cells are among the targets for arsenic exposure, and that the inhibition of GSIS (glucose stimulated insulin secretion) by the methylated metabolites may be the key mechanism of arsenic-induced diabetes [91].

### Arsenic-induced liver disease

About arsenic induced liver disease (including hepatomegaly, cirrhosis, portal tract fibrosis, etc), it was found that arsenic can mediated oxidative stress, activating c-Jun N-terminal kinases (JNK) and p38 mitogen-activated protein kinases (MAPK) pathway to induce hepatic apoptosis. Likewise, it can also induces hepatic cell death by upregulation of pro-apoptotic proteins [92–94]. Moreover, histological examination of the livers has revealed a consistent finding of portal tract fibrosis by arsenic exposure [95]. Individuals exposed more frequently to arsenic suffer from cirrhosis, which is considered to be a secondary effect of damage to the hepatic blood vessels [96]. Chronic arsenic exposure in animals can also produce liver endothelial cell damage, which subsequently damages parenchymal cells [97]. All these studies clearly revealed that exposure to arsenic are associated with hepatomegaly, hepatic fibrosis and cirrhosis. Hence, oxidative stress, apoptosis, and up regulation of apoptosis related proteins are the prospective target sites for arsenic induced hepatotoxicity [98].

### Arsenic-induced cardiovascular diseases (CVD)

With respect to cardiovascular diseases (CVD) in human population, it suggested that not only arsenic but also genetic, environment and nutritional factors necessary for development of this disease. Epidemiological studies have shown that arsenic ingestion through food or water may have serious effects on the human cardiovascular system including heart damage (i.e., myocardial depolarization, hypertrophy of the ventricular wall, cardiac arrhythmias), vascular damage (Blackfoot disease), ischemic heart disease, cerebrovascular diseases, and hypertension [21, 99, 100].

It is believed that vascular endothelial cells play a pivotal role in arsenic-induced cardiovascular diseases. Increased activity of nicotinamide adenine dinucleotide phosphate (NADPH) oxidase enzymes (NOX) presents in the plasma membrane of vascular endothelial cells and vascular smooth muscle cells are associated with vascular disease or hypertension [101]. Indeed, arsenic has the rapid stimulatory effects on these enzymes to generate reactive oxygen species (ROS) [102] and nitric oxide (NO) to form peroxynitrite, a strong oxidant, implicated in the up regulation of inflammatory mediators [103]. Correspondingly, arsenic can also interacting with G-protein coupled receptors (GPCR) to initiate signal amplification schemes regulating NOX-dependent redox signaling [101]. In addition, generation of ROS by arsenic increases the expression of atherosclerosis related genes such as heme oxygenase-1 (HO-1), monocyte chemo-attractant protein-1 (MCP-1), and interleukin-6 (IL-6) and thus its exposure promotes the attachment, penetration, and migration of monocytes in vascular smooth muscle cells [104]. Along with this, endothelial cell activation/dysfunction by arsenic produces several inflammatory molecules such as soluble intercellular adhesion molecule-1 (sICAM-1), soluble vascular adhesion molecule-1 (sVCAM-1), MCP-1 related to the atherosclerotic lesions [105]. On the other hand, our group has found that trivalent MMA^III^ has unique toxic effect on cellular differentiation of embryonic stem cells to cardiomyocytes [106]. All these observed associations suggest a potential pathway underlying the effect of arsenic exposure-related atherosclerosis leading to CVD.

### Arsenic-induced nephrotoxicity and neurotoxicity

It is well known that arsenic exposure can causes the nephrotoxicity in mammals. Prevalent of chronic kidney disease (CKD) is inversely associated with the sum of inorganic and methylated arsenic concentrations in urine, and particularly with inorganic arsenic concentrations. Prospectively, inorganic plus methylated arsenic concentrations in urine, especially monomethylarsonate and dimethylarsinate concentrations were positively associated with incident of nephrotoxicity [107]. Among the three trivalent arsenicals (iAs^III^, MMA^III^ and DMA^III^), MMA^III^ have shown more potent cytotoxic effect on human urothelial cells *in vitro* [108]. Moreover, recent evidence also indicates that developmental arsenic exposure impacts renal carcinogenesis in stem cells. Tokar et al. reported that arsenic targeting the multipotent stem cells and they found that long term exposure of arsenic can causes carcinogenic transformation of stem cells, implying that the targeting of stem cells during early stages of life may be the key for the development of arsenic-induced cancers in kidney [109]. Furthermore, an interesting result has been found when rats exposed to DMA^V^ results in development of carcinogenesis in bladder [110, 111]. However, neither the proximate carcinogen nor the mechanism of action underlying cancer development has been identified. However, the metabolites found in urine from rats those exposed to DMA^V^ includes methylated oxoarsenicals (e.g., DMA^III^ and TMAO^V^) [112–114] and methylated thioarsenicals (e.g., dimethylmonothioarsinic acid; DMMTA^V^) potential proximate carcinogens [115].

In addition to above adverse toxic effect of arsenic on different organs, it has also found that low concentrations of methylated arsenic species (e.g., MMA^III^) could inhibit sensory neuron and skeletal muscle formation. This may provide a mechanism for the low birth weight and changes in neurological function seen in arsenic exposed epidemiological studies [116, 117].

### Effects on vital proteins/enzymes

It was found that arsenic-induced disturbance in cellular energy related nucleotides may also result in redox state alterations [118]. Moreover, increased the secretion of inflammatory factors and IL-8, TNF-α, and TGF-α expression have also been observed to be induced by arsenic exposure [119]. It is well known that arsenic species in particular trivalent methylated arsenic intermediate metabolites have high binding affinity for the cysteine residues of proteins, however, arsenic has also been recognized for inhibiting the functioning of various transcriptional co regulators, proteins and enzymes [120–123].

Arsenicals can have both genotoxic and non-genotoxic mechanisms of action, and also serve as a complete carcinogen [124]. Additionally, it has proposed that arsenic species can influence the function of the DNA repair enzymes, as in particular the trivalent arsenic compounds including iAs^III^ and its methylated metabolites (MMA^III^ and DMA^III^) have been recognized for inhibiting DNA repair [83, 125–127]. Recently, it has been indicated that iAs^III^ can bind to the zinc finger motifs in recombinant protein which having 3 or more than 3 cysteine residues, eventually altering the function of DNA repair proteins and/or mechanisms [128, 129]. The methylated trivalent arsenic metabolites showed more efficient interacting ability with the cysteine containing repair proteins like the one involved in nucleotide excision repair (NER) as compared to their precursor; iAs^III^ [83]. Moreover, xeroderma pigmentosum group A protein (XPA), which is recognized for its important role in NER mechanism, has recently shown to be affected by arsenicals [130].

Notably_,_ regarding the trivalent arsenicals interaction with Zinc finger domain of the XPA (containing Zn^2+^) at normal physiological condition, it has been found that only methylated metabolites; MMA^III^ and DMA^III^ (but not iAs^III^) have stronger binding affinity to zinc finger proteins [119]. In fact, the studies that have shown binding of iAs^III^ to zinc finger peptides were mostly performed using apo-Zn finger proteins [128, 131], thus, more studies are needed to evaluate that whether iAs^III^ can also bind to Zn finger peptides or proteins (i.e., with Zn^2+^) even under normal physiological conditions.

Likewise, metallothionein (MT), a cysteine rich protein, is commonly known for protecting against toxicity of heavy metals. Thus, many researchers have also investigated the effect of iAs^III^ on MT, they found that iAs^III^ is able to induce the MT expression *in vivo* and *in vitro* [127, 132]. Additionally, it has suggested that iAs^III^ can probably bind to the C-terminal cysteine cluster of metal-activated transcription factor 1 (MTF1) and thereby, induce the transcription activity [133]. On investigating the binding interaction of the three trivalent arsenicals (i.e., iAs^III^, MMA^III^ and DMA^III^) with the metallothioneins, MMA^III^ was found to have the highest binding capacity among the three arsenic species [134], suggesting that the arsenic-induced cytotoxicity might probably have some association with arsenic-binding to proteins.

### Arsenic induced skin lesions

Skin symptoms of arsenic exposure are hallmarks of the early stages of the arsenic poisoning in the population exposed to arsenic through drinking water. Nonmalignant skin lesions have a short latency period and may appear within a few years of exposure. Arsenic exposure related skin symptoms include: melanosis (diffuse and spotted), keratosis (diffuse and spotted), leucomelanosis (rain drop pigmentation), and hyperkeratosis. Melanosis a dark pigmentation on the skin and keratosis is an area of small corn like elevations (0.4-1 cm in diameter), usually appears as bilateral thickening of the palms and soles, are found at the primary stage of arsenic-induced all dermatological manifestations [135, 136], leuko-melanosis and hyperkeratosis in the second stage and ultimately may turn to skin cancer such as Bowen's disease, basal cell and squamous cell carcinoma [11, 137, 138].

Chromosomal abnormality, oxidative stress and altered growth factors provide a basis for some of these alterations [139, 140]. Arsenic triggers ROS production in the skin and induces accumulation of unfolded proteins in ER which causes ER stress [141–143]. Under stress condition, the chaperone GRP78 dissociates from ER membrane resident sensors PERK, IRE1α and ATF6α [144], which lead to their phosphorylation or proteolysis-dependent activation. Activated IRE1α functions as a nuclease and splices XBP-1, that can initiates many signaling pathways and finally activates UPR target genes in the skin. In addition, arsenic-induced ROS can also activate p38 MAP kinase and its downstream protein MAPKAPK-2 [145]. Thereby, UPR and p38 MAP kinase are considered to be responsible for the onset of inflammation in the skin [146].

### Arsenic-induced carcinogenicity

The carcinogenic potential of arsenic was recognized over 110 years ago by Hutchison, who observed an unusual number of skin cancer occurring in patients treated for various diseases with medical arsenicals [147]. The International Agency for Research on Cancer (IARC) and the US Environmental Protection Agency (EPA) has classified arsenic as a carcinogen based on epidemiological studies [7, 148]. Arsenic carcinogenesis is major concern affecting numerous organs of human. Epidemiological studies carried out in different countries have demonstrated an evident causal relationship between environmental, occupational, and medical exposure of millions of people worldwide to inorganic arsenic and increased risks of cancer of the skin, lungs, urinary bladder, kidney, prostate, liver and other sites [149–152]. Although the exact molecular mechanism of arsenic carcinogenicity is not well understood, progression of carcinogenesis by arsenic is considered to be associated with intracellular signal transduction, transcription factors activations and the abnormal genes expression.

On the other hand, promotion of oxidative stress by arsenic compounds is one of the main causes for arsenic-induced carcinogenicity [153, 154]. It has been reported that several arsenic metabolites (e.g., DMA^V^ and TMAO^V^) cause an elevation of 8-hydroxydeoxyguanosine by generation of ROS, stimulates cell proliferation and induces carcinogenicity [155, 156]. Additionally, arsenic induces MAPK signal transduction pathway that alter various gene expression profile via AP-1 and NF-kB and causes cancer [157]. Arsenic causes focal adhesion kinase activation which regulates various downstream signaling pathways (e.g., EGFR, ERK, cadherins) of carcinogenesis [158]. Moreover, arsenic can damage DNA or changing methylation patterns, stimulate angiogenesis, dysregulating cell cycle, blocking physiological apoptosis and causes skin, bladder and lung cancer [159–161]. Although the precise cellular mechanism by which arsenic induces cancer is still unknown, collectively oxidative stress, genotoxic effects, growth factors expression, loss of DNA repairing mechanisms are proposed to be the possible modes of carcinogenic action of arsenic.

## ANTICANCER ACTIVITIES OF INTERMEDIATE METHYLATED METABOLITES OF ARSENIC

Arsenic compounds have widely been used as traditional medicines in Mongolian, Chinese and Tibetan for the treatment of various diseases such as psoriasis, syphilis, and rheumatosis for over two thousand years [162]. Approximately, 60 different arsenic preparations have been developed and distributed during the lengthy history of this agent and many of these preparations are still in use. Although the IARC and the U.S EPA have classified arsenic as a known human carcinogen, several arsenic compounds have been recently rediscovered and formulated to treat different diseases.

Arsenic compounds such as Darinaparsin (DAR), namely DMA^III^(GS), GSAO [4-(N-(S-glutathionylacetyl) amino) phenylarsonous acid] and Trisenox (As_2_O_3_) are in clinical trials of U.S. Food and Drug Administration for the treatment of cancers such as leukemia, lymphomas and solid tumors [163, 164]. In this section, we will describe about the metabolism and possible effects of these arsenic compounds in cancer treatments.

### Darinaparsin

Darinaparsin (DAR) a novel organic form of arsenic that was rationally designed to provide more efficient effects to kill tumor cell through increase intracellular arsenic concentration and increased apoptosis. It is composed of dimethylated arsenic (an inorganic arsenic metabolite) and glutathione (Figure 1C). This combination of DAR in the biological environment is thought to be inert but in fact, DAR has shown to be effective with clinically acceptable doses *in vitro* against leukemia, myeloma, lymphoma, and various solid tumor cell lines [165, 166]. Based on promising results from the *in vitro* experiments, the investigation on DAR was extended to preclinical and clinical studies. In case of hematologic cancers, refractory solid tumors, multiple myeloma, and hepatocellular carcinoma the efficiency, safety and pharmacokinetics of DAR have been evaluated [167–169].

Phase II study of DAR in relapsed or refractory Hodgkin's and non-Hodgkin's lymphoma, including patients presenting with peripheral T-cell lymphoma (PTCL), provides preliminary evidence that DAR is active and well tolerated [170]. Over expression of the multi-drug resistant protein ABCC1 in PTCL may be the determinant factors for the decrease sensitivity to ATO, while cytotoxicity of organic arsenic species DAR is not altered in these cells because of ABCC1 is not the transporter of DAR, which resulting in increase DAR concentration in cells [171].

Additionally, the cellular uptake of DAR is different from other arsenic compounds. The presence of intracellular GSH was shown to affect the uptake of DAR. It has already been reported that DAR enter the cells in the form of DMA^III^ at low concentrations of exogenous glutathione or in the absence of it. Before uptake into the cells, DAR is suggested to be processed by the enzyme γ-glutamyl transpeptidase (γ-GT) on the cell surface to S-(dimethylarsenic) cysteine (DMA^III^(Cys)) [172], finally the multiple cystine/cysteine importers transport DMA^III^(Cys) into the cells, whereas no transporter has been yet identified that could transport intact DAR.

**Figure 4:**
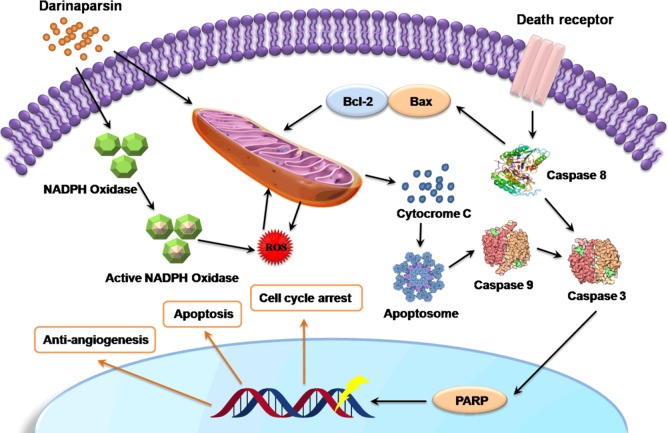
Effects of Darinaparsin (DAR) on Cellular Functions Production of intracellular ROS is the key events of DAR mediated apoptosis. Generally, it occurs through either by the activation of NADPH oxidase complex or direct effect on mitochondrial membrane potential. Abnormal mitochondrial functions are associated with the release of cytochrome c resulting in the activation of apoptosome, which causes initiation of apoptosis. The ultimate result is cell cycle arrest, induction of apoptosis and anti-angiogenic activity.

DAR has known to have multifarious and complex mechanistic pathways to show its effects. Figure 4 schematically represents the possible anticancer mechanistic pathways of DAR. Generally, it induces G2/M cell cycle arrest and apoptosis in tumor cells primarily through disruption of mitochondrial functions by inducing cleavage of caspase 8 and 9 (Figure 4). Additionally production of ROS can also initiate the apoptotic pathway and usually DAR initiates two major pathways for ROS generation, namely, NADPH oxidase activation and disruption of the mitochondrial transport chain. In fact, DAR activates cytosolic subunits of NADPH oxidase complex p67PHOX and p47PHOX, resulting in the translocation of NADPH oxidase to the membrane and produces ROS, which disrupt mitochondrial membrane potential and release of Cytochrome c [173]. This leads to the activation of apoptosome that ends into cellular apoptosis [165].

### GSAO

**Figure 5:**
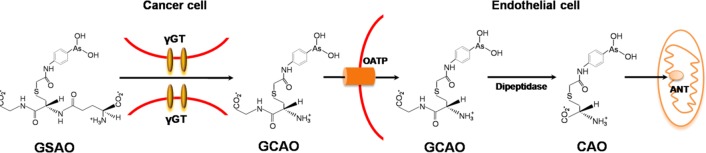
Molecular mechanism of GSAO GSAO is activated by tumor γGT at the cell surface, and the following GCAO is transported across the plasma membrane by the organic anion transporting polypeptide (OATP). Additionally, GCAO is possibly further processed to CAO by dipeptidase in the cytosol and finally reacts with ANT of the inner mitochondrial membrane.

GSAO (4-(N-(S-glutathionylacetyl)amino) phenylarsonous acid) is a conjugate of glutathione and phenylarsonous acid as shown in Figure 1D. In vitro and in vivo experiments where tumor metastasis relies heavily on neovascularization GSAO exhibited potential anti-cancer effects [174–177]. More specifically, GSAO targets a key mitochondrial transporter in tumor endothelial cells and metabolism of GSAO at the cell surface is required to exert its anti-mitochondrial effect. During biotransformation, the γ-glutamyl residue of GSAO is cleaved at the cell surface by γ-glutamyl transpeptidase and 4-(N-(S-cysteinylglycylacetyl)amino) phenylarsonous acid (GCAO) forms and then enters the cell through an organic ion transporter (Figure 5). Moreover, it probably be converted to 4-(N-(S-cysteinylacetyl)amino)phenylarsonous acid (CAO) by dipeptidases, and CAO is responsible for pharmacological effects of GSAO. For instance, CAO reacts with mitochondrial adenine nucleotide translocase (ANT) and inactivates via cross-linking two of the three matrix facing cysteine thiols. Proper functioning of ANT is essential for cell viability, targeting this protein in angiogenic endothelial cells is a powerful means of blocking angiogenesis and cell proliferation, finally resulting in cell death. The possible mechanism of action of GSAO is summarized in Figure 5.

The mechanism of action of GSAO described here implies that GSAO should be more effective against γ GT-positive tumors. Notably, tumors of the breast, prostate, colon, liver, and ovary express γ GT, whereas soft tissue tumors tend not to express this enzyme [178]. Metabolism of GSAO by tumor endothelium γ GT is able to produce high concentrations of GCAO, which will block tumor angiogenesis and tumor growth. Based on the different laboratory findings, GSAO is currently being trialed in a Phase I/IIa dose escalation study in patients with solid tumors. As phase I clinical trials have not been completed, their side effects and toxicities in humans are largely unknown. Additionally, hematological cancers do not rely on new blood vessel formation for proliferation, therefore, it is only suitable for patients with solid tumors refractory (resistant) to standard therapy.

### Trisenox (As2O3)

Trisenox (As_2_O_3_) one of the most effective novel anticancer agents used for the treatment of APL that received approval from the U.S. FDA in 2000 as anticancer agent. The molecular mechanism of As_2_O_3_ in front-line therapy has been largely studied among all arsenic compounds [179, 180]. Generally, in human after administration of As_2_O_3_, it is hydrolyzed to iAs^III^ and then metabolized into trivalent methylated intermediate metabolites; MMA^III^ and DMA^III^ by AS3MT, and the oxidized MMA^V^ and DMA^V^ are considered to be the end products of As_2_O_3_ [39]. However, it is still indistinct regarding the anticancer effects of its methylated metabolites (i.e., MMA^III^, MMA^V^, DMA^III^ and DMA^V^). Therefore, it needs to be identified the precise mechanism of the effects of As_2_O_3_ and its metabolites to find out the actual benefits from this vital agent for the treatment of APL or other types of cancer. It has been found that As_2_O_3_ is able to induce differentiation of APL cells at low doses (0.25~0.5μM), while on the other hand high doses of As_2_O_3_ (1~2μM) could induce apoptosis [181]. Wang et al (2004) made an attempt to determine the arsenic species in urine of APL patients receiving As_2_O_3_ treatment by HPLC-ICP MS analysis [182]. Interestingly, they found that MMA^III^, DMA^III^, MMA^V^ and DMA^V^ were detected in the urine of these patients, particularly, methylated pentavalent arsenic species being the major arsenic species, followed by only a minor amount of trivalent methylated arsenic species found in urine samples. Similar results were also obtained from the blood sample of APL patients after As_2_O_3_ treatment, they found the order of these arsenicals concentrations (free form) in plasma to be as MMA^V^ > DMA^V^ > iAs^III^ > iAs^V^ and no trivalent methylated arsenicals observed, indicating that As_2_O_3_ can be rapidly transformed into methylated arsenic species in body, and most of trivalent methylated arsenicals considered to be binding with proteins [58]. Interestingly, As-binding proteins have confirmed in plasma and RBCs of APL patients by HPLC column after receiving As_2_O_3_ treatment, and found that arsenicals in trivalent form bound to protein in blood [183]. Based on above findings, it was considered that arsenic metabolites (e.g., MMA^III^ and DMA^III^) might exert vital role in APL patients after treatment with As_2_O_3_. Thus, the role of arsenic methylated metabolites in the clinical efficacy against APL or other cancer treatments should be examined.

Hikita et al. has used pentavalent organic arsenic species (e.g., MMA^V^, DMA^V^, TAO^V^, AsB, AsC) to determine their anticancer effects on T-lymphoblastoid leukemia cells [184]. Unexpectedly, the pentavalent organic arsenic compounds showed no inhibitory effects on the leukemia cell proliferation as compared to iAs^III^. Indeed, these results clearly indicated that pentavalent organic arsenicals have no appreciable anticancer effects,. Additionally, trivalent iAs^III^, MMA^III^ and DMA^III^ have also been used to determine the anticancer effects against human myeloid leukemia HL-60 cells [185], and methylated arsenic species were found to be more potent in decreasing the cell survival as compared to its precursor iAs^III^. However, HL-60 cells have also exhibited to be relatively resistant to organic arsenic species than compared to other cell lines. Lower uptake and higher efflux may be the possible reason for cellular resistance to arsenicals. In order to understand the molecular mechanism of HL-60 cells resistance to arsenic, we interested to determine the expression of MRP transporters. Interestingly, MRP1 was found to be over-expressed in HL-60 cells, which confer resistance to inorganic and organic arsenic compound (i.e., iAs^III^ and MMA^III^). Additionally, when HL-60 cells were pretreated with MK571(inhibitor of MRP1), the cells became highly sensitive to arsenic treatment, suggesting that after blocking the transporter which responsible for arsenic efflux could increase the cellular sensitivity to arsenic treatment [186].

**Figure 6:**
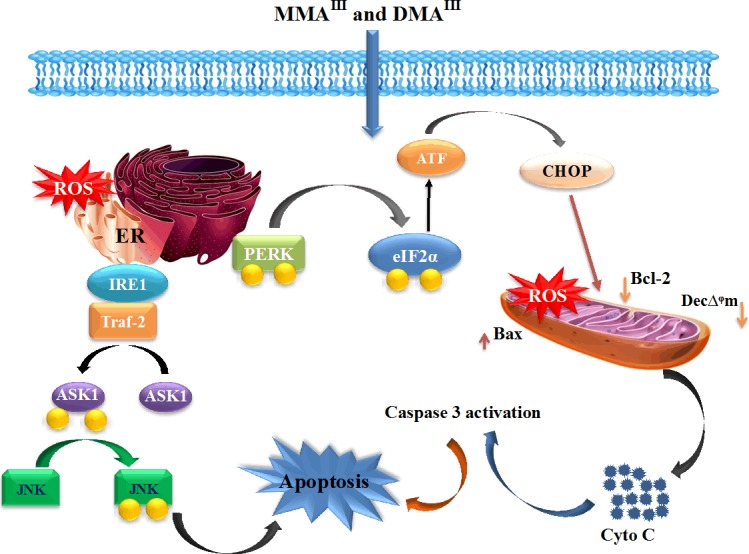
Toxicity and Anticancer Effects of MMA **III** and DMA**III**. MMA^III^ specifically inhibit the activity of complexes II and IV, which results in electron leakage from complex I and III causing the generation of ROS in mitochondria, resulted in induction of mitochondrial dysfunction and release Cyt c, finally inducing apoptosis through caspase-3 dependent pathway. DMA^III^ predominantly target ER, stimulating the activation of PERK whose activation leads to the phosphorylation of eIF-2α, translation initiation of ATF4 and CHOP, resulting in induction of apoptosis. Similarly, DMA^III^-induced ER-stress also activate IRE1, this activation of IRE1 further forms a complex with TRAF2 recruiting ASK1, which in turns activates JNK to induce apoptosis.

On the other hand, MMA^III^ found to be specifically inhibit mitochondrial electron transport chain (ETC). Namely, MMA^III^ inhibit the activity of complexes II and IV, which results in electron leakage from complex I and III causing the generation of ROS in mitochondria, resulted in induction of mitochondrial dysfunction and apoptosis [187]. Differently from the MMA^III^, DMA^III^ was found to predominantly target the endoplasmic reticulum (ER), stimulating the activation of PERK whose activation leads to the phosphorylation of eIF-2α, translation initiation of activating transcription factor 4 (ATF4) and CHOP, resulting in induction of apoptosis [188, 189], suggesting that the two methylated trivalent arsenic species use different mode of actions to showed their activity (Figure 6). Likewise, ER-stress can also activate inositol-requiring enzyme 1 (IRE1) which belongs to ER transmembrane receptors, this activation of IRE1 further recruits and forms a complex with TNF-receptor-associated factor 2 (TRAF2) recruiting apoptosis-signal-regulating kinase (ASK1) which in turn activates JNK [190], while arsenic mediated activation of JNK is recently found to be important for the anticancer effects of arsenic [191, 192].

Therefore, iAs^III^ and its trivalent methylated metabolites were evaluated for their potential effects. It was found that methylarsine oxide (i.e., MMA^III^) followed by iododimethylarsine (i.e., DMA^III^) significantly induced growth inhibition and cellular apoptosis in NB4, K562 human leukemia, lymphoma cell lines, and primary culture of chronic lymphocytic leukemia cells when compared to iAs^III^ [192]. However, unlike iAs^III^, methylarsine oxide could not induce degradation of Promyelocytic leukemia-retinoic acid receptor alpha (PML-RARα) fusion protein in APL cells. This study suggested that though arsenic methylated metabolites could not induce cellular differentiation or PML-RARα fusion protein degradation in APL cells, but still are potent inducer of cellular apoptosis as compared to their precursor iAs^III^ and therefore, the arsenic metabolites might be used in leukemia and lymphoma treatments [193].

**Figure 7:**
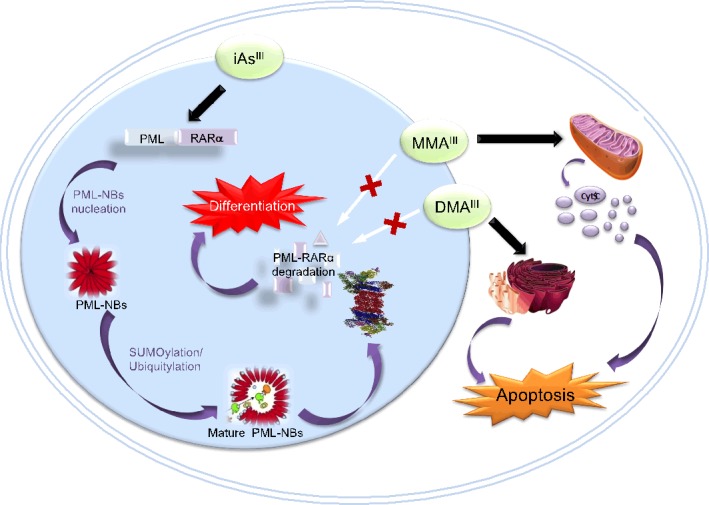
Molecular Mechanisms of iAs **^III^** and its Trivalent Methylated Metabolites **(i.e., MMA**^III^ **and** DMA^III^) **Induced Cell Differentiation and Apoptosis in APL Cells**. Arsenic trioxide in hydrolyzed form, i.e., iAs^III^ may cause formation of PML-RARα protein multimers, initiate the nucleation of PML-NBs, enhanced SUMOylation/ubiquitination, resulting degradation of PML-RARα fusion protein by ubiquitin/proteasome pathway, finally promoting cell differentiation leading to clinical remission. On the other hand, MMA^III^ and DMA^III^ have shown no effect on induction of PML-RARα protein degradation and APL cell differentiation. Conversely, they have strong effect on induction of apoptosis in APL cells through mitochondria and ER-dependent pathways.

Very recently, it has been found that As_2_O_3_ induced APL cells differentiation occurs as a result of direct binding of As_2_O_3_ to cysteine residues of zinc fingers in the RING finger-B Box-Coiled Coil (RBCC) domain of PML-RARα fusion protein, which results in enhanced SUMOylation/Ubiquitination and then degradation of PML-RARα fusion protein, promoting cell differentiation leading to clinical remission. However, in *in vitro*, among trivalent arsenic species (i.e., iAs^III^, MMA^III^ and DMA^III^), the methylated MMA^III^ has shown much stronger binding affinity for various proteins including Zinc finger proteins or PML-RARα fusion proteins as compared to other two arsenic species (Figure 7) [131], but methylated arsenicals were incapable of inducing protein degradation, suggesting that arsenic induced PML-RARα fusion protein degradation is complicated. Although two methylated arsenic species could not degrade PML-RARα fusion protein in NB4 cells, these compounds have exhibited much more potent effect on induction of apoptosis, as schematically drawn in Figure 7. These are the underlying principle for the evaluation of the arsenic intermediate metabolites against solid tumors, APL and/or non-APL malignancies.

## CONCLUSIONS AND FUTURE PERSPECTIVES

The precise and appropriate metabolic pathways involved in arsenic metabolism are still a matter of question, and are of enormous value in understanding the mechanisms of arsenic induced toxicity and in determining the implication of arsenic in therapeutic advances. The actual arsenic metabolic pathway would be helpful in elucidating the probable role of different arsenic species in relation to toxicities along with the uses of arsenic as a drug.

On the other hand, arsenic as a traditional medicine, inappropriate use of arsenic may poison people, while its appropriate application cures some cancer types and saves lives. The revival of arsenic by its application in treating APL is a unique story in cancer research. The therapeutic magnitude of arsenicals deliberated over here may also be enlightening in near future with respect to the precious uses of arsenicals as medicines for the treatment of diseases. However, there are necessities to further investigate the effects of these metabolites and other arsenic species to assess whether these underlying principles can be interpreted into important clinical outcomes through carry out potential preclinical and clinical studies.

## Abbreviations

As_2_O_3;_: Arsenic trioxide
APL: Acute promyelocytic leukemia
GSH: Glutathione
iAs^III^: Arsenite
SAM: S-adenosylmethionine
MMA^III^: Monomethylarsonous acid
MMA^V^: Monomethylarsonic acid
DMA^III^: Dimethylarsinous acid
DMA^V^: Dimethylarsinic acid
iAs^V^: Arsenate
TMAO^V^: Trimethylarsine oxide
AS3MT: Arsenic (+3 oxidation state) methyltransferase
As(GS)_3_: Arsenic triglutathione
MMA(GS)_2_: Monomethylarsenic diglutathione
DMA^III^(GS): Dimethylarsenic glutathione
AQPs: Aquaglyceroporins
MRP: Multidrug resistance proteins
GST: Glutathione S transferase
GSTP1: Glutathione S-transferase π1
(RBCs): red blood cells
Cys: Cysteine
GLUT4: Glucose transporter type 4
PDK-1: 3-phosphoinositide-dependent kinase-I
mTOR: Mammalian target of rapamycin
GSIS: Glucose stimulated insulin secretion
JNK: Jun N-terminal kinases
MAPK: mitogen-activated protein kinases
ROS: Reactive oxygen species
NOX: Nicotinamide adenine dinucleotide phosphate (NADPH) oxidase enzymes
NO: Nitric oxide
GPCR: G-protein coupled receptors
HO-1: Heme oxygenase-1
MCP-1: Monocyte chemo-attractant protein
NER: Nucleotide excision repair
XPA: xeroderma pigmentosum group A protein
MT: metallothionein
MTF1: Metal-activated transcription factor 1
ER: Endoplasmic reticulum
ATF4: Activating transcription factor 4
IRE1: Inositol-requiring enzyme 1
TRAF2: TNF-receptor-associated factor 2
PML: Promyelocytic leukemia
RARα: Retinoic acid receptor alpha
RBCC: RING finger-B Box-Coiled Coil
